# Characteristics of Hospitalized Adults With Recurrent Urinary Tract Infection Due to Extended Spectrum Beta-Lactamase Producing Escherichia coli in a Tertiary Center in Saudi Arabia

**DOI:** 10.7759/cureus.33054

**Published:** 2022-12-28

**Authors:** Badriah H Alanazi, Najla Alrasheed, Jamila A Alonazi, Mohammed Alqahtani, Amjad Alseraya, Rajkumar Rajendram, Majed Alsheikh, Abdullah Bawazir, Fayzah Dugashim, Bayan Albdah

**Affiliations:** 1 Department of Medicine, King Abdulaziz Medical City, Riyadh, SAU; 2 Department of Internal Medicine, King Abdulaziz Medical City, Riyadh, SAU; 3 College of Medicine, King Saud Bin Abdulaziz University for Health Sciences, Riyadh, SAU; 4 Department of Pathology and Laboratory Medicine, King Abdulaziz Medical City, Riyadh, SAU; 5 Section of Biostatistics, Department of Biostatistics and Bioinformatics, King Abdullah International Medical Research Center, Riyadh, SAU

**Keywords:** risk factor, esbl uti, esbl e.coli, esbl, uti

## Abstract

Background: The increase in extended-spectrum β-lactamase (ESBL) producing microbes in recent years represents a major challenge.

Aim: To study the risk factors for urinary tract infections (UTIs) caused by ESBL-producing *Escherichia coli* in patients requiring hospitalization for treatment.

Materials and method: Electronic health records were used to identify 616 inpatients over the age of 18 who had UTI symptoms and/or signs and an ESBL-producing *E. coli* strain cultured on urine culture between January 1 and December 31, 2018. The electronic health care records of these patients were searched to identify those patients with previous UTIs due to an ESBL-producing *E. coli* grown on urine culture. Patients with cancer or those taking prophylactic antibiotics or immunosuppression were excluded.

Result: Risk factors for the acquisition of ESBL-producing *E. coli* included male sex (P = 0.0032), age over 66 years (P < 0.0001), renal stones (P = 0.0021), urology intervention within six months of presentation (P = 0.0360), pressure sores (P = 0.0002), feeding tubes (P = 0.0076), and urinary catheter (P = 0.0023). Comorbidities (e.g., diabetes mellitus and duration of antibiotic therapy were not associated with an increased risk of recurrence of ESBL-producing *E. coli* UTI (P = 0.4680, P = 0.3826, respectively).

Conclusion: Antimicrobial stewardship programs may have reduced the development of antimicrobial resistance in *E. coli*. However, the recognition of risk factors for UTI caused by ESBL-producing *E. coli* may facilitate the early detection of high-risk cases and guide treatment decisions. This can improve patient outcomes while decreasing the length of the hospital stay.

## Introduction

Urinary tract infection (UTI) is the second most common cause of community-acquired infection and healthcare-associated infection in clinical practice worldwide [1,2]. The pathogens are usually Gram-negative bacilli (GNB), and *Escherichia coli* is the most frequently identified microorganism.

Bacteria can evolve highly specialized mechanisms of resistance to commonly used antibiotics [3]. One resistance mechanism of particular concern is the production of an extended-spectrum β-lactamase (ESBL). Bacteria producing ESBL remain susceptible to cephamycins (e.g., cefoxitin and cefotetan) and carbapenems (e.g., meropenem, imipenem, and ertapenem) [4].

Expression of an ESBL confers resistance to many of the first-line antibiotics commonly used to treat UTIs. These include penicillin, third-generation cephalosporins (e.g., ceftazidime, cefotaxime, and ceftriaxone), and monobactams (e.g., aztreonam) [1,4]. Treatment failure can occur if one of these classes of drugs is used to treat a severe UTI due to an ESBL-producing organism. Thus, the spread of ESBL-producing bacteria has become a serious threat to global public health.

One retrospective study of ESBL-producing *E. coli* was conducted at King Khalid University Hospital, Riyadh, Saudi Arabia, from June 2009 to June 2011 [1]. Of 339 adults and children with culture-proven *E. coli* UTI, 113 (33.3%) had an ESBL producer [1]. In children, *E. coli* that did not produce an ESBL was more common [1]. Women were most commonly infected with an ESBL-producing *E. coli* [1]. Other risk factors identified by Al-Otaibi et al. [1] included patients with underlying renal disease or a renal transplant (p=0.017), vesicoureteral reflux in children (p=0.044), and surgical intervention [1].

However, few studies have assessed the characteristics of patients admitted with urinary tract infections caused by ESBL-producing GNB, and the literature on risk factors is conflicting. We, therefore, performed a retrospective analysis of the risk factors for UTI caused by ESBL-producing *E. coli* at a tertiary care hospital in a low-prevalence country.

## Materials and methods

Study design and area and settings 

A retrospective cross-sectional study was performed at King Abdulaziz Medical City, Riyadh (KAMC).

Identification of study participants

All inpatients over 18 years old with a diagnosis of ESBL UTI and an ESBL-producing *E. coli* grown on urine culture between January 1 and December 31 of 2018 were identified from electronic medical records (EMR). EMRs of these patients were searched to identify those with previous UTIs due to an ESBL-producing *E. coli* grown on urine culture. Patients with cancer or those taking prophylactic antibiotics or immunosuppression were excluded.

Data collection process 

Data were extracted from EMR created between January 1, 2018 and December 31, 2018. Besides standard demographics (i.e., age, sex), information was obtained about comorbidities, the sites of any pressure sores, the site of insertion (i.e., urethral, suprapubic, nephrostomy), and type (i.e., permanent, intermittent, inserted only in hospital) of an indwelling urinary catheter, the results of imaging of the renal tract (e.g., renal ultrasound), and the history of the antibiotic regimen used to treat previous UTI due to ESBL-producing E.coli (i.e., medication, dose, frequency, and duration). Recurrent UTI due to ESBL-producing *E. coli* is defined as two or more proven cultures of active and symptomatic UTIs in 90 days period.

Data analysis 

Data were analyzed using the statistical program SAS (version 9.4, SAS Institute, North Carolina, USA), data are presented as the frequency with a percentage for categorical variables, Fishers Exact test or the Chi-squared test were used to assess the association between categorical variables. All statistical tests were considered significant at P < 0.05.

## Results

At KAMC in 2018, symptomatic UTI caused by ESBL-producing *E. coli* was identified in a total of 616 patients admitted for treatment [age range 20 years to 114 years; median age 66 years; interquartile range (43 years to 78 years); 189 males (30.68%)] with recurrent UTI due to ESBL-producing *E. coli*. The demographic profile, comorbidities, antibiotic use, and imaging of the study population are summarized in Table 1.

**Table 1:** Demographic data, comorbid disease, antibiotic therapy, and recurrent urinary tract infection with extended spectrum beta-lactamase producing E. coli. *Statistically significant P-value.

	Risk factors	Subgroups	% of patients (1 time of ESBL UTI in 90 days) (N)	% of patients (2-5 times of ESBL UTI in 90 day) (N)	P-value
Demographic	Age	≤66 years	55.36 (248)	38.10 (64)	0.0001*
		>66 years	44.64 (200)	61.90 (104)	
	Sex	Female	72.77 (326)	60.12 (101)	0.0032*
		Male	27.23 (122)	39.88 (67)	
Comorbidity	Diabetes	No	47.10 (211)	43.45 (73)	0.4680
		Yes	52.90 (237)	56.55 (95)	
	Hypertension	No	45.31 (203)	41.07 (69)	0.3633
		Yes	54.69 (245)	58.93 (99)	
	Stroke	No	79.91 (358)	73.81 (124)	0.1244
		Yes	20.09 (90)	26.19 (44)	
	Pressure sores	No	93.74 (419)	83.33 (140)	0.0002*
		Yes	6.26 (28)	16.67 (28)	
	Tube feeding	No	85.71 (384)	76.19 (128)	0.0076*
		Yes	14.29 (64)	23.81 (40)	
Disease of the renal tract	Urology intervention within 6 months	No	96.21 (431)	91.67 (154)	0.0360*
		Yes	3.79 (17)	8.33 (14)	
	Renal stones	No	95.09 (426)	87.50 (147)	0.0021*
		Yes	4.91 (22)	12.50 (21)	
	Prostate disease	No	89.04 (398)	84.52 (142)	0.1303
		Yes	10.96 (49)	15.48 (26)	
	Chronic kidney disease	No	85.71 (355)	76.19 (129)	0.5818
		Yes	14.29 (93)	23.81 (38)	
	Previous renal tract ultrasound	No	42.41 (190)	25.60 (43)	0.0001*
		Yes	57.59 (258)	74.40 (125)	
Antibiotic	Prior antibiotic	No	14.73 (66)	17.86 (30)	0.3826
		Yes	85.27 (382)	82.14 (138)	
	Type of antibiotic	Carbapenem	27.23 (104)	34.06 (47)	0.0689
		Cephalosporin	9.95 (83)	12.32 (17)	
		Fluoroquinolone	23.56 (90)	23.19 (32)	
		Nitrofurantoin	21.99 (84)	10.14 (14)	
		Other	9.16 (35)	10.87 (15)	
		Piperacillin and tazobactam	8.12 (31)	9.42 (13)	
	Frequency of antibiotic	Twice daily	47.12 (180)	54.35 (75)	0.0004*
		Daily	6.02 (23)	2.17 (3)	
		Four times a day	26.18 (100)	12.32 (17)	
		Three times a day	20.68 (79)	31.16 (43)	
	Duration of antibiotic	7-14 days	75.85 (289)	78.99 (109)	0.4831
		<7 days	24.15 (92)	21.01(29)	
	Route	Intravenous	38.74 (148)	50 (69)	0.0265*
		Oral	61.26 (234)	50 (69)	
Urinary catheter	Urinary catheter in situ	No	78.52 (351)	99.07 (111)	0.0023*
		Yes	21.48 (96)	33.93 (57)	
	Duration in situ	<1 month	60.82 (59)	33.33 (19)	0.0014*
		>1 month	39.18 (83)	66.67 (83)	
	Type	Intermittent	32.98 (31)	26.32 (15)	0.4668
		Permanent	67.02 (63)	73.68 (42)	

Demographic data

Approximately half of the study population (304, 49.35%) were over 66 years old at presentation to the hospital. This subgroup was at greater risk of recurrent ESBL *E. coli* UTI (61.90%) than those 66 years old or less (P < 0.0001). Male patients (39.88%) were also at higher risk of recurrent ESBL-*E. coli* UTIs (P = 0.0032).

Comorbid disease, indwelling urinary catheters and risk of recurrent ESBL *E. coli* UTI 

The risk of recurrent UTI with ESBL-producing *E. coli* was not significantly affected by diabetes mellitus (P = 0.4680), hypertension (P = 0.3633), chronic kidney disease (P = 0.5818), stroke (P = 0.1244), or prostate disease (P = 0.1303). However, the risk of recurrent UTI with ESBL-producing *E. coli* was significantly increased by renal stones (P = 0.0021), pressure sores (P = 0.0002), tube feeding (P = 0.0076), and urology intervention within the six months prior to presentation (P = 0.0360).

The risk of recurrent ESBL-*E. coli* UTU was increased in patients with an indwelling urinary catheter (P = 0.0023). The risk of recurrence was greater in those patients who had required urinary catheterization for more than one month (P = 0.0014). However, there was no difference between intermittent catheterization and the use of a permanent catheter (P = 0.4668).

Renal ultrasound was more likely to have been performed in those patients who had recurrent UTI caused by ESBL-producing *E. coli* (P = 0.0001). However, the findings of renal tract ultrasound (enlarged prostate, hydronephrosis, normal, stone, tumor, and others) were not statistically significant (P = 0.0752).

Antibiotic therapy and risk of recurrent ESBL *E. coli* UTI

The risk of recurrent UTI due to ESBL-producing *E. coli* was not affected by antibiotic use (P = 0.3826) or the duration of antibiotic use (7-14 days vs. under 7 days; P = 0.4831). The specific choice of antibiotic (carbapenem, cephalosporin, fluoroquinolone, nitrofurantoin, piperacillin, tazobactam, and others) did not affect recurrence (P = 0.0689).

However, the risk of recurrence was influenced by the frequency of antibiotics (P = 0.0004). The risk was higher in those who received antibiotic therapy twice a day (BID) or three times a day (TID). The risk of recurrence was less in patients who received antibiotics daily or four times a day (QID). The risk of recurrent UTI caused by ESBL-producing *E. coli* was higher in patients receiving intravenous antibiotics than that in patients treated with an oral antibiotic (P = 0.0265).

## Discussion

The current paradigm for antibiotic stewardship in the treatment of UTI prioritizes the use of first-line therapies such as trimethoprim and nitrofurantoin [5]. However, these agents are often ineffective against ESBL-producing *E. coli*. It is therefore important to determine the risk factors for these multidrug-resistant organisms.

Risk factors for recurrent ESBL *E. coli* UTI

Seven risk factors for recurrent ESBL *E. coli* UTIs were identified in the present cohort. These were men over the age of 66 who had had urology intervention within the previous six months, such as renal stones, pressure sores, feeding tubes, or a urinary catheter. These findings are consistent with previous studies.

Age and Male Sex 

Age and male sex are well-recognized risk factors for UTI due to ESBL-producing *E. coli* [6-8]. Increasing age is associated with dysfunction of the immune system and increased susceptibility to infections [9]. Furthermore, elderly men are more likely to develop prostatitis or a catheter-associated UTI.

Urinary Tract Catheterization and Other Urological Interventions

Catheterization of the urinary tract increases the risk of ESBL *E. coli* UTI [10-12]. The observations of the present study confirm this and reiterate the importance of limiting the duration of catheterization. The presence of a urinary catheter for more than one month was associated with a greater risk of recurrent ESBL-producing *E. coli* UTI than if the catheter had been present for less than one month. Urinary catheters may become coated with a biofilm that acts as a reservoir for microorganisms [13].

Similar to catheterization, other interventions in the urinary tract also compromise the action of antibiotics and host defense mechanisms [13]. Thus, consistent with previous studies [6,14], urological intervention within the six months prior to presentation was also associated with an increased risk of UTI due to the presence of ESBL-producing *E. coli* in the present cohort. Further studies are required to define the selection of peri-procedural antibiotic prophylaxis in patients at risk of ESBL-producing *E. coli* who undergo urological intervention.

Renal Ultrasound, Renal Disease and Calculi

Patients in the present cohort who had a renal ultrasound prior to presentation were more likely to have a recurrent UTI with an ESBL-producing *E. coli*. While the findings of the renal ultrasound did not correlate with risk, the risk of recurrent UTI due to ESBL-producing *E. coli* was increased in patients with renal stones but not chronic kidney disease. In contrast, Al-Otaibi et al. [1] reported that UTIs due to ESBL-producing *E. coli* were more common in patients with renal disease (P = 0.017) but less common in patients with renal stones [1].

Renal calculi increase the risk of infection due to ESBL-producing GNB [15]. Urinary tract stones and obstructive uropathy are more frequently observed in *E. coli* infections [15]. Ureteric obstruction can cause urinary stasis and renal dysfunction [15]. Stasis increases bacterial adherence, facilitating their invasion of the urogenital epithelium [15]. Urinary calculi also provide a surface for bacterial proliferation. The apparent contradiction between our observations and those of Al-Otaibi et al. [1] may reflect differences in the severity of renal disease in the two cohorts.

Pressure Sores and Tube Feeding

Tube feeding and the presence of pressure sores were also associated with a significantly increased risk of recurrent UTIs with ESBL-producing *E. coli*. This finding is consistent with previous reports [10,14]. The presence of pressure sores or feeding tubes indicates poor functional status. Such patients often have urinary or bowel incontinence and are more likely to be exposed to cross-contamination of bacteria from the bowel to the urinary tract [14].

Factors that did not increase risk of recurrent ESBL *E. coli* UTI

When considering empirical antibiotic therapy for UTI prior to the availability of culture results, it is important to determine the risk of ESBL-producing GNB. It is therefore important to determine the factors that do not increase the risk of ESBL-producing *E. coli*.

Diabetes Mellitus

Aswan et al. reported that the prevalence of ESBL-producing *E. coli* was significantly higher in patients with diabetes (P=0.001). However, in the present cohort, the risk of recurrent UTI due to ESBL-producing *E. coli* was not affected by diabetes. Previous studies have also reported similar results [1,16,17]. This may reflect differences in the control of diabetes and the severity of glycosuria in these cohorts.

It is important to note that none of the patients in the present cohort were treated with sodium glucose-like transporter 2 (SGLT-2) inhibitors. Some studies suggest that the risk of UTI is increased by these agents [18]. Further studies are required to clarify the risk of UTI due to ESBL-producing GNB in patients treated with an SGLT-2 inhibitor.

*Hypertension* 

In the present cohort, hypertension was not associated with an increased risk of UTI due to ESBL-producing *E. coli*. While previous studies have reported similar findings [1], the data on the relevance of hypertension in this setting are conflicting. For example, Maria et al. reported that hypertension was associated with ESBL-producing *E. coli* [19]. However, as older patients are more likely to have increased blood pressure and ESBL-producing *E. coli*, hypertension may have been a confounder in previous studies.

Prostate Disease

In the present cohort, prostate disease was not associated with UTI due to ESBL-producing *E. coli*. While previous studies have reported similar findings [10], data on the relevance of prostate disease in this setting are conflicting. For example, Azap et al. reported that prostatic disease is associated with ESBL-producing *E. coli* [11].

As described above, urethral catheterization and urological procedures increase the risk of UTI [10-14]. The presence of these well-recognized risk factors is relatively uncommon in the general population. However, their incidence increases dramatically with prostate volume, which also increases with age [20]. This prostate disease may have been a confounder in previous studies.

Prior Antibiotic Use 

Prior antibiotic use is the most frequently identified risk factor for UTI due to ESBL-producing *E. coli* [21,22]. However, in the present cohort, prior antibiotic use was not associated with ESBL-producing *E. coli*. Studies published in 2014 and 2016 reported similar findings [21,22]. Antimicrobial stewardship programs have refined the management of asymptomatic bacteriuria and UTI [5]. This approach may have reduced the impact of antimicrobial therapy on the development of antibiotic resistance in GNB, including *E. coli* [5].

Limitations

The limitations of the present study include being a retrospective study and the lack of a control group. Thus, potential confounding factors may have influenced statistical analyses. Furthermore, the present study did not detect any association between specific antibiotic usage and ESBL-producing *E. coli*. This may be because only inpatients were included in the present cohort. Thus, it is not possible to determine whether a hospital stay or antibiotic treatment affected the risk of ESBL-producing *E. coli*. In addition, the data for our study were collected from a single-center study; therefore, this study’s findings cannot be generalized to the regional or national level.

## Conclusions

The prevalence of ESBL infection in patients and healthcare professionals is influenced by several risk factors. Most studies agree that factors such as hospital admission and previous antibiotic use are important risk factors. However, the relevance of other factors (e.g., sex, age, renal disease, and other comorbidities) is less clear. The present study identified risk factors for UTI due to ESBL-producing *E. coli* in a tertiary care center in Riyadh, Saudi Arabia. Male sex, age over 66 years, renal stones, urology intervention within six months of presentation, pressure sores, feeding tubes, and a urinary catheter were associated with an increased risk of UTI due to ESBL-producing *E. coli*, while comorbidities (e.g., diabetes mellitus) and the duration of antibiotic therapy were not associated with an increased risk of recurrence of an ESBL-producing *E. coli* UTI. Antimicrobial stewardship programs may have reduced the incidence of antibiotic resistance in *E. coli*. However, the identification of patients at high risk of UTI due to ESBL-producing *E. coli* is required to guide the choice of empirical therapy.
